# Shared Environment Effects on Children’s Emotion Recognition

**DOI:** 10.3389/fpsyt.2019.00215

**Published:** 2019-04-11

**Authors:** Rotem Schapira, Hillary Anger Elfenbein, Meirav Amichay-Setter, Carolyn Zahn-Waxler, Ariel Knafo-Noam

**Affiliations:** ^1^The Mofet Institute, Tel-Aviv, Israel; ^2^Levinsky College of Education, Tel-Aviv, Israel; ^3^Washington University, St. Louis, MI, United States; ^4^The Hebrew University of Jerusalem, Jerusalem, Israel; ^5^University of Wisconsin-Madison, Madison, WI, United States

**Keywords:** empathy, emotion recognition, shared environment effect, individual differences, childhood

## Abstract

Empathy is relevant to many psychiatric conditions. Empathy involves the natural ability to perceive and be sensitive to the emotional states of others. Thus, emotion recognition (ER) abilities are key to understanding empathy. Despite the importance of ER to normal and abnormal social interactions, little is known about how it develops throughout childhood. We examined genetic and environmental influences on children’s ER *via* facial and vocal cues in 344 7-year-old twin children [59 monozygotic (MZ) and 113 same-sex dizygotic (DZ) pairs], who were part of the Longitudinal Israeli Study of Twins. ER was assessed with the child version of the Diagnostic Assessment of Nonverbal Accuracy. For both facial and vocal cues of emotion, twin correlations were not higher for MZ twins than for DZ twins, suggesting no heritability for ER in this population. In contrast, correlations were positive for both types of twins, indicating a shared environmental effect. This was supported by a bivariate genetic analysis. This pattern was robust to controlling for twins being of the same sex and age. Effects remained after controlling for background variables such as family income and number of additional siblings. The analysis found a shared environmental correlation between facial and vocal ER (*r*_c_ = .63), indicating that the shared environmental factors contributed to the overlap between vocal and facial ER. The study highlights the importance of the shared environment to children’s ER.

## Introduction

Empathy, the ability to perceive and be sensitive to others’ emotional states (1), is relevant to many psychiatric conditions (2). Emotion recognition (ER) abilities are relevant to feeling empathy for others (3), specifically cognitive empathy (4), the ability to recognize and understand the emotions of others (5). Despite the importance of ER for social interaction and functioning (6–8), individuals vary markedly in ER ability (9, 10). Our research addresses the origin of these individual differences. Specifically, we investigated genetic and environmental effects on children’s recognition of emotion from facial and vocal cues using data from seven-year-old twins.

Childhood may be a unique developmental period for ER, with important developmental advances such as the ability to take others’ perspective, which contribute to better understanding of emotions and social interactions (11–13). Nevertheless, little is known about how ER develops throughout childhood [e.g., Ref. (12)]. Most of the studies on the contribution of the family context to children’s emotional development did not directly address ER but focused on related skills, such as children’s emotion understanding (14). Many environmental factors may influence social development, including parental socialization, peer influence, teachers, school, and culture (15–17). Much research has also addressed the roles of socioeconomic status (SES) and sex on ER development (18). There is evidence that children with low SES show difficulties in terms of their overall emotional development (19) and, particularly, in ER (20), and in a meta-analysis of 215 studies (21), females had a small but reliable advantage in ER tasks.

Although theoretical and empirical research suggests that individual differences in empathy are affected by both genetic and environmental factors (22), only little research has examined genetic and environmental effects on ER. Studies estimating genetic and environmental influences on empathy have typically relied on the classic twin design, which compares monozygotic (MZ) and dizygotic (DZ) twins (23). Higher similarity between MZ than DZ twins indicates genetic influence (*heritability*), while twin similarity that is not higher for MZ twins cannot be accounted for by their genetic relatedness and is attributed to the *shared environment*. Finally, dissimilarity between family members despite their genetic and environmental relatedness indicates the influence of the *nonshared* environment and measurement error.

We found only two past studies of genetic effects on ER. Studying 10-year-old twins (*N* = 250 pairs), Lau and colleagues (24) found modest and largely nonsignificant genetic effects on recognition of specific emotions from facial expressions, and a strong (75% of the variance) genetic effect on a global factor estimated across emotions. The second study (25) also examined facial emotion recognition in a larger sample of twins (*N* = 957 individuals) in a wide range of ages, 9 to 17. The findings show a significant genetic effect (34%–57%) for the recognition of six basic emotions. Only modest evidence was found for shared environment effects (1%–12%), controlling for age and sex. Nonshared environmental effects accounted for the remaining variance in both studies.

Substantial changes occur in ER during middle childhood (11, 12). For example, age plays an important role in the emotion comprehension process, and cognitive nonverbal factors are predictors of 3- to 10-year-olds’ emotion comprehension (26). Moreover, past work has shown that the relative importance of genetic and environmental effects changes with age [for a meta-analysis on empathy, see Ref. (22)]. It is therefore important to extend the results to younger samples. Based on past work, we expected to find a genetic effect and nonshared environmental influence on children’s ER in our 7-year-old sample. Additionally, based on the above evidence, we examined the role of SES and sex in ER.

Past work (24) focused on facial expressions. Importantly, recent studies have shown the importance of vocal cues to accurate ER (27). Vocal cues improve nonverbal communication and ER in social situations [e.g., Refs. (28, 29)]. While preschool children tend to rely more on facial expressions during social interaction, school-aged children (ages 7–12) rely on both facial expression and tone of voice (30). The importance of vocal cues to emotion, then, calls for studying them in addition to facial cues. Our study, therefore, expands the scope of ER by testing both facial expressions and vocal tone. In addition, studying both kinds of cues in the same design enables an investigation of the origin of the association between understanding vocal and facial cues to emotion. Overlapping genetic effects on facial and vocal ER would indicate a global cross-modality genetic tendency. In contrast, overlapping environmental effects will indicate that similar environmental forces promote (or hinder) development of ER across modalities.

## Methods

### Participants

A total of 344 Jewish Israeli children (52% male, 59 MZ pairs and 114 same-sex DZ pairs) participated in the Longitudinal Israeli Study of Twins (LIST) (31) at the age of 7 years (90.05 ± 3.87 months). Children were observed performing a variety of tasks in the lab. Each child was tested separately by a different experimenter from his or her twin to avoid any bias effects. Written informed consent was obtained from the participants’ parents.

### Measures

#### Emotion Recognition Measure

We used the child version of the Diagnostic Assessment of Nonverbal Accuracy Scale-2 (DANVA-2) (7, 32, 33). Children watched 24 different pictures of children’s faces one at a time and classified each as angry, happy, fearful, or sad. Similarly, they are required to identify the different emotions in 24 recordings of oral speech, where the words themselves are emotionally neutral (the same sentence is used: “I am going out of the room now but I will be back later”). The DANVA has been extensively used and well validated in child samples [e.g., Refs. (34–36)]. Inter-item reliability of the items yielded Cronbach’s *α *= .70. Facial and vocal cues correlated positively (*r* = .30, *p* < .001) and were analyzed separately as well as summed into an overall ER score.

### Demographic Data

Mothers reported demographic data, including number of additional siblings and SES. SES was indexed by family income, asking parents to rate their income relative to the given national average using a scale ranging from 1 “a lot below” to 5 “a lot above” the average (*M* = 3.26, SD = 1.26).

### Analyses

We performed descriptive analyses with SPSS (version 25). Genetic analyses were performed using the Mx structural equation modeling software (37). Mx was specifically designed to analyze twin data, estimating the relative contribution of additive genetic (A), shared environment (C), and nonshared environment and error (E) effects on individual differences. We also used a bivariate extension of the twin design using the correlated factors model (38), which estimated the ACE components for each modality separately, as well as the associations between the genetic and environmental components contributing to each modality.

## Results

**Table 1** presents descriptive statistics and correlations between MZ and DZ twins for overall ER and for facial and vocal cues separately. Results showed positive correlations in both MZ and DZ twin pairs. Correlations were not higher for MZ twins, indicating no heritability for ER in this population. Instead, positive correlations for both DZ and MZ twins indicate that at least part of the individual differences in these measures is associated with shared environmental factors. Although the DZ correlation was somewhat higher than the MZ correlation, this difference was not significant (Fisher’s test of independent correlations, *z* = −1.85). Similar correlation patterns were found for both facial and vocal cues of emotion.

**Table 1:** Twin correlations and genetic/environmental influences on ER.

	Correlations		Variance component estimates proportion (95% CIs)			Model fit indices	
	MZ twins	DZ twins	Genetics	Shared environment	Nonshared environment	AIC	BIC
**Total ER**	.35**	.49**	.00 (.00–.24) –	.44(.24–.56) [.44(.32–.56)]	.55 (.44–.68) [.56 (.44–.68)]	263.99 261.99	−396.18 −398.76
*M* (SD)	24.32 (6.12)	25.48 (5.85)					
**Facial**	.29**	.35**	.00 (.00–.38) –	.32 (.03–.45) [.32 (.18–.45)]	.68 (.52–.82) [.68 (.55–.82)]	540.72 534.72	−781.26 −788.99
*M* (SD)	15.48 (4.40)	16.41 (4.11)					
**Vocal**	.21	.47**	.00 (.00–.19) –	.38 (.19–.50) [.38 (.24–.50)]	.63 (.50–.76) [.63 (.50–.76)]	540.72 534.72	−781.26 −788.99
*M* (SD)	8.84 (3.37)	9.03 (3.02)					

The second line for each variable represents the best-fitting model after dropping the genetic effect estimated at 0.00. The parentheses portray the estimates from the full ACE model including the genetics, whereas the brackets contain the estimates from the model without the genetics. AIC, Akaike information criterion, calculated in comparison to the full ACE model. BIC, Bayesian information criterion. CI, confidence intervals. MZ, monozygotic, DZ, dizygotic, same-sex twins. *p < .05, **p < .001

**Figure 1:**
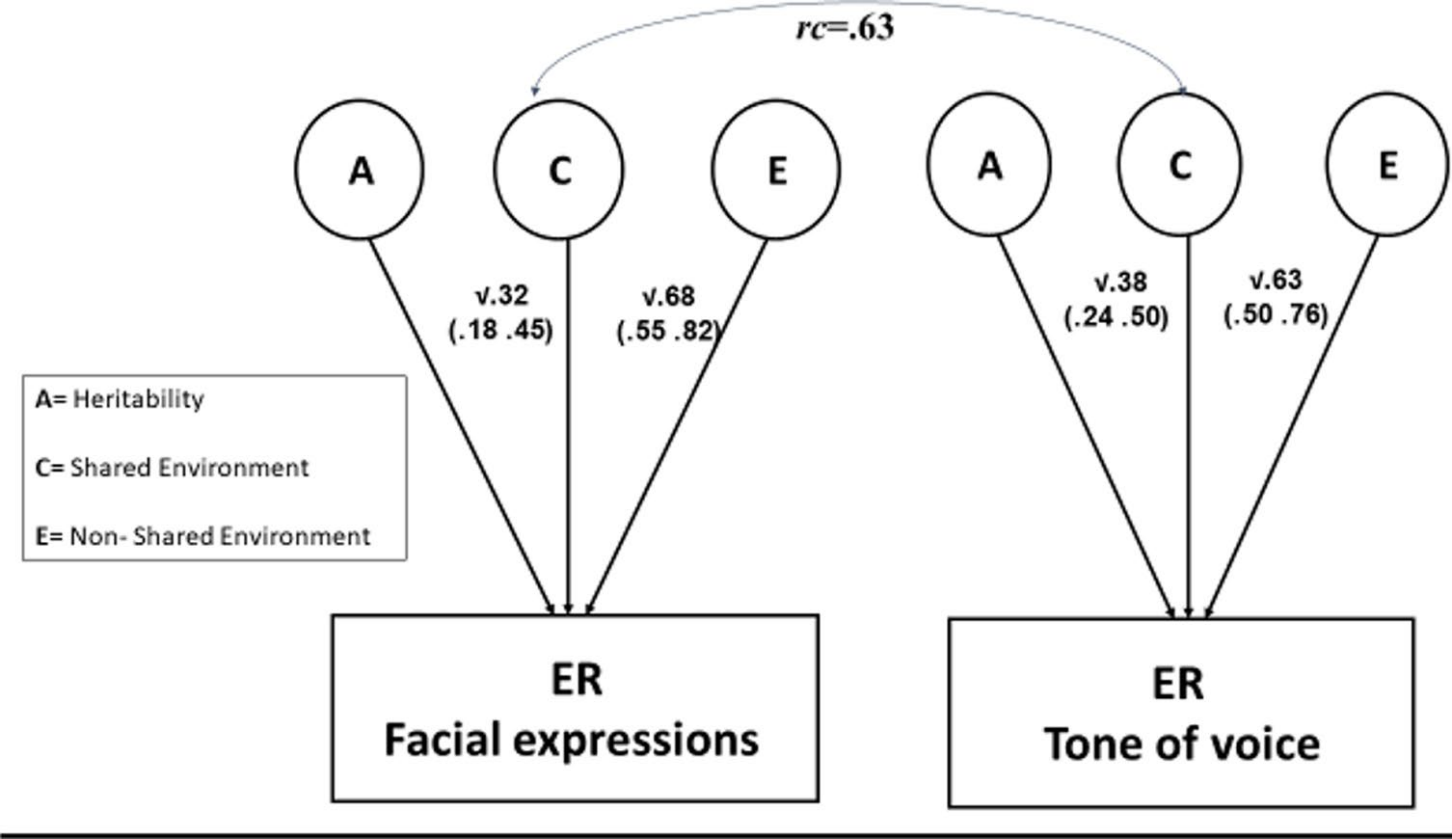
The bivariate model: genetic and environmental influences on ER.

Our first genetic analysis fitted a univariate genetic model to the overall ER scores. As the genetic effects were estimated at zero, they were dropped from the model without affecting model fit [Δχ^2^ (*df* = 1) = 0.00]. Thus, the model without a genetic effect (CE) was preferred over the less parsimonious full model (ACE). The shared environment effect accounted for 44% of the variance, and the remaining variance was accounted for by the nonshared environment effect and error (**Table 1**). We estimated genetic and environmental contributions to facial and vocal ER, as well as the association between these two variables. We fitted a bivariate genetic model to the data. Again, genetic effects were estimated at 0 and could be dropped from the model without worsening fit [Δχ^2^ (*df* = 3) = 0.00], and the more parsimonious CE model was preferred. The shared environment component accounted for 32% and 38% of the variance in facial and vocal ER, respectively. The bivariate genetic analysis indicated that the correlation between facial and vocal ER reflected a shared environmental correlation between these variables [*r*_c_ = .63, 95% confidence interval (CI) = .34–.94], with little correlation between the nonshared environment components (*r*_e_ = .09, 95% CI = −.05 to .25) (**Figure 1**). The shared environment effect accounted for 73% of the correlation between facial and vocal ER (based on the product of *r*_c_ and the nonsquared standardized shared environment path coefficients) with the rest of the association accounted for by the nonshared environment effect.

**Table 1** presents fit indices for the univariate and bivariate CE models. These model fit indices reflected the pattern in which similarity between the DZ twins is greater than the MZ twins, which is not expected in the CE model given that the shared environment is estimated as affecting siblings growing up together similarly regardless of genetic similarity.

Age variation in months within our sample correlated with facial ER (*r* = .23, *p* < .01) and vocal ER (*r* = .22, *p* < .01). In addition, girls performed better than boys in facial [*t* (376) = −4.43, *p* < .004, *D* = .46] and vocal ER [*t* (375) = −1.72, *p* < .001, *D* = .18], in line with previous work (21, 30). It was therefore important to account for age and sex differences among twin pairs and to verify that the shared environment effects found in the study go beyond the effects of twins sharing their sex and age. We thus calculated a new ER variable, partialing out the effects of sex and age in a regression analysis. Controlling for sex and age, the results still held (*r*MZ = .23, *r*DZ = .46), showing no genetic effect. That is, age and sex did not inflate twin correlations and could not account for the shared environment effects estimated.

ER correlated modestly with demographic variables such as greater SES (*r* = .14, *p* < .05) and fewer additional siblings (*r* = −.16, *p* < .001). However, as was the case with age and sex, follow-up analyses showed that the presence of the shared environment effects was beyond twins’ sharing these variables. Specifically, analysis of scores residualized for number of siblings, SES, sex, and age did not substantially change the results, including the lack of genetic effect. The shared environment component was estimated at 23% (CI = .07–.38) and 38% (CI = .23–.51) of the variance in facial and vocal ER, respectively, and a shared environmental correlation accounted for the association between these variables (*r*_c_ = .46, CI = .06–.88).

## Discussion

This study examined genetic and environmental influences on children’s ER, for the first time adding vocal to facial cues of emotion. Our results indicate that individual differences in ER by 7-year-old children are accounted for by shared and nonshared environmental variables. Moreover, the association between facial and vocal cues reflected mainly overlapping shared environmental effects. This study highlights the importance of the environment to children’s ER.

Shared environment effects suggest that the family milieu plays an important role in the development of children’s ER (16), although the exact process needs further research. Moreover, the bivariate analysis indicated that shared environmental factors largely account for the association between vocal and facial ER. Across species, the social environment provides a place for training and learning about the emotional world, helped by social factors such as contact and familiarity (39). Research has shown that it is easier for individuals to identify others’ emotion expressions from their own cultural in-group (40, 41). In addition, culture may be influential through stereotypical displays found in various media (42). These archetypes provide an opportunity to gain exposure and learn about the emotional social world. Going to the family level, it is possible that differences among families in the expression of emotions affect children. These shared experiences may affect the ability to understand emotions in a stereotypical and nonexhaustive way, increasing similarity between siblings being exposed to similar events in their family.

A large portion of the individual differences in ER in this study were attributed to nonshared environment effects (43). Nonshared effects are child specific and can include life events such as illness and relationships with family and peers. The study focused on 7-year-olds who, in the Israeli context, are already attending school. School may serve as an important source of nonshared environmental influences, as twins are exposed to a variety of different peers and often different classrooms. Communicating with diverse children exposes children to others’ varied emotional states. This can then enable further peer experience and expertise in ER, which may contribute to the nonshared environment effects on ER.

We did not find any genetic effects, while most past twin studies on empathy and related variables found meaningful genetic effects and little evidence for shared environmental effects (22, 36, 44, 45). One possibility is that our use of a test method vs. the more commonly used questionnaire methods led to lower heritability estimates, as was found for other variables (e.g., parenting) (46). However, past work on facial expressions did find genetic effects (25). Another possibility is that the lack of genetic effects reflects the younger age of our sample as compared to past studies (5, 24). Indeed, heritability increases with age for several traits (47, 48), including empathy (22), in longitudinal studies using the same method across ages (49).

Thus, further longitudinal research might support the increase in heritability with age. One way in which heritability might increase with age is through evocative gene–environment correlation processes, in which the child’s genetically influenced traits increasingly affect the environment, which in turn influences the developing person, leading to an increase in heritability with age (50–52). In addition, the absence of heritability should be interpreted in light of the possibility that genetic effects are moderated or influenced by environmental factors, known as gene–environment interactions.

The consistency of results for facial and vocal cues strengthens our findings. Similarly, controlling for the effects of sex and age, the results still held and showed no genetic effect while highlighting the importance of the shared environment. We note that the sample is not large and focused only on 7-year-olds. Future studies should increase the sample size and examine different ages and development over time.

We tested children’s ER directly using the DANVA (29, 53). As the two were examined in separate rooms, they could not affect one another during testing. At the same time, the DANVA emphasizes very specific facial expressions that could be influenced by family background. Future work should also use subtler cues to express emotion in a more complex and less stereotypical way. In addition, in real life, emotion is perceived through an integration of visual and auditory cues (27), and thus, a pathway for future work is to study vocal and facial cues jointly in the same stimulus.

Our findings contribute to understanding the development of emotion recognition, a core aspect of empathy. They call for in-depth investigation of environmental factors involved in psychiatric disorders characterized by difficulties in emotional recognition.

## Ethics Statement

The study was approved by the Ethics Committee of the Hebrew University of Jerusalem, Israel.

## Author contributions

RS, AK-N, HA, and CZ-W contributed to the conception and design of the study. RS organized the database. AK-N performed the statistical analysis. RS wrote the first draft of the manuscript. AK-N, HA, MA-S, and CZ-W wrote sections of the manuscript. All authors contributed to manuscript revision and read and approved the submitted version.

## Funding

This study was conducted with the support of MOFET Institute, the John Templeton Foundation, and Levinsky College of Education in Tel-Aviv, Israel.

## Conflict of Interest Statement

The authors declare that the research was conducted in the absence of any commercial or financial relationships that could be construed as a potential conflict of interest.
